# Radiation Exposure from Fluoroscopy during Hip Arthroscopy

**DOI:** 10.1055/s-0039-3400278

**Published:** 2019-11-21

**Authors:** Roberto Seijas, Jordi Català, Miguel Ángel Cepas, Andrea Sallent, Oscar Ares, David Barastegui

**Affiliations:** 1 Department of Orthopedic Surgery, Instituto Cugat, Hospital Quirón, Barcelona, Spain; 2 Garcia-Cugat Foundation, Barcelona, Spain; 3 Department of Anatomy, Universidad Internacional de Catalunya, Barcelona, Spain; 4 Department of Radiology, Instituts Guirado, Barcelona, Spain; 5 Hospital Quirón, Barcelona, Spain; 6 Department of Orthopedic, Hospital Vall d'Hebron, Barcelona, Spain; 7 Department of Orthopedic and Trauma, Hospital Clínic Barcelona, Spain; 8 Mutualitat Catalana de Futbolistes, Barcelona, Spain

**Keywords:** femoroacetabular impingement, hip arthroscopy, fluoroscopy, radiation exposure

## Abstract

**Objective** Hip arthroscopy for femoroacetabular impingement treatment is a procedure that is not exempted from complications. The most common complications are related to the arthroscopic portals and the traction system. The use of fluoroscopy helps in hip arthroscopy; however, the radiation exposure is a risk that has not yet been studied.

**Materials and Methods** A retrospective study with 100 arthroscopies was performed. Surgical indication in all cases was femoroacetabular impingement. Surgical times and radiation exposure during the procedure had been recorded and reviewed for the present study.

**Results** A mean of 138.20 cGy cm ^2^ radiation exposures was observed per patient and procedure for a mean time of radiation exposure of 0.36 minutes. These values are much lower than the values described as being at risk by the nuclear security commissions.

**Conclusions** Radiation exposure in a hip arthroscopy due to femoroacetabular impingement is in margins well below the limits at risk for the patient.

Femoroacetabular impingement (FAI) has sustained a growing increase in its knowledge and thus in the number of surgeries designed to treat it, as well as a technic and scientific development in the past years. 1

Despite the arthroscopic procedures have progressed to improve the access to the central and peripheral areas of the hip, 2 there is an existing risk of injuries, especially when the surgical instruments go over the labrum or the femoral head's cartilage. The use of fluoroscopy helps reducing these risks, however, with radiation exposure for the patient. Moreover, the risk of radiation exposure may be even greater in patients with a more difficult access to the joint. 3

In our country, the risks of radiation exposure are regulated according to the recommendations by the European Atomic Energy Community (Euratom) according to the regulations by the Nuclear Safety Commission, and proposed by the Spanish Ministries of Economy, Home Affairs, Health and Social Services, Employment and Social Security, and Defense. 4 5 6

Due to the more complex pathologies that are being treated in hip arthroscopy, 1 we considered evaluating the radiation exposure that patients receive and know the grade of safety in this field.

Our hypothesis is that radiation received by patients undergoing a hip arthroscopy due to FAI entails a radiation dose within the safety limits established and is therefore reasonable and safe.

## Materials and Methods

A retrospective study with 100 patients was performed, evaluating the radiation exposure of patients undergoing a hip arthroscopy in our center. Inclusion criteria included diagnosis of FAI, age over 18 years old, and no previous pathology of the hip. One single institution surgical and anesthetic team performed all surgeries with the same radiologic technician.

An informed consent was obtained from all participants for the present study.

### Surgical Procedure

**Fig. 1:**
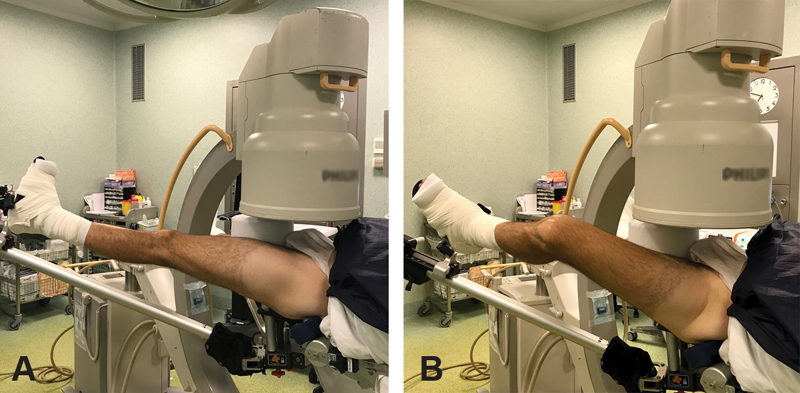
( **A** , **B** ) Positioning of the fluoroscopy C-arm in anteroposterior and axial views of the left hip.

The procedures were performed in supine position with intradural anesthesia. Hip arthroscopy was performed in all cases in supine position with a specific hip positioning system (Smith and Nephew, Memphis, TN). The same fluoroscopic C-arm (Philips BV Pulsera, Philips Medical System; Eindhoven, The Netherlands) was used in all surgeries. Longitudinal traction was used in the operative extremity to distract the hip for introduction of the instruments, with foam-padded boots and perineal-padded post to protect skin or nerve injuries due to the traction applied. 7 The C-arm fluoroscopic arm is brought to the operative field and placed over the operative hip, between the patient's legs, offering an anteroposterior (AP) view of the joint ( Fig. 1 ). The hip is then gently distracted under fluoroscopic guidance to obtain ∼10 mm distance between the acetabulum and femoral head. A spinal needle is used for the first portal (anterolateral), 3 and then the guide is removed to pass the Nitinol guidewire (Disposable Hip Pac, Smith & Nephew, MA), placing an obturator-cannula over. Then, the obturator is removed to introduce the 70° arthroscope (VideoArthroscope 70° HD DirectView, Smith & Nephew, MA). The distal anterior and anterior portals are done under arthroscopic vision to perform the labral suture and treatment of acetabular and femoral cartilage lesions. Once the central space has been approached and treated, traction is removed to easily reach the peripheral space of the hip and perform the cervico-cephalic femur osteoplasty under fluoroscopic guidance in AP view and with flexion-rotation of the hip for an axial view ( Fig. 1A and B ).

### Radiation Exposure

Evaluation of radiation-absorbed dose by the patient was studied by the dose area product (DAP) (cGy cm ^2^ ), which is the total radiation energy delivered to the tissues, and the effective dose (E, mSv), related directly to radiation-associated risk (stochastic risk). Conversion factor is 0.2 x DAP = mSv. Twenty millisieverts are equal to ∼1,000 chest X-rays. If 1,000 adult patients are irradiated with 20 mSv, it is estimated that at least one of them could die of radio-induced cancer. 8 9

Accumulative skin dose (Gy) represents the deterministic (direct) effects on the skin. It is defined as the dose that the patient's skin will absorb if the X-ray beam irradiates always the same skin area. Based on DAP it can be approximately worked out. Over 3,000 mGy (3 Gy) should be a dangerous situation that requires a clinical follow-up. 8 9

High-dose procedure radiation was considered when 5% of cases exceed DAP of 300 cGy cm. 2 8 9

The skin dose is hard to calculate precisely due to the different variables that are checked out: use of filtrations, different angulations in taking pictures, variability in the field of view's size, etc.; however, skin dose is calculated approximately using the following formula:

Maximum skin dose (cGy) = DAP (cGy cm ^2^ ) / area (cm ^2^ ) × 1.3. 8 9

Normal skin dose for fluoroscopy is 15 to 45 cGy cm ^2^ , high if it is over 50 to 150 cGy cm ^2^ and limit with 200 cGy cm ^2^ .

### Statistical Analysis

The descriptive statistical analysis involved the use of tables of absolute frequencies for qualitative variables and measures (mean, median) central tendency and dispersion (standard deviation [SD]) for continuous quantitative variables.

All statistical analysis was performed using SPSS for Mac, version 20.

## Results

In a retrospective study, 100 consecutive patients with FAI who were undergoing a hip arthroscopy were included. Table 1 shows epidemiological values of the cases studied.

**Table 1:** Epidemiologic data

	Study group
**Patient count (** ***n*** )	100
**Age (y), mean ± SD**	41.35 ± 10.52
**Gender**	
**Male,** ***n*** **(%)**	65 (65%)
**Female,** ***n*** **(%)**	35 (35%)
**BMI, mean ± SD**	25.17 ± 4.14

Abbreviations: BMI, body mass index; SD, standard deviation.

The type of FAI and the surgical details of labrum management are described in Table 2 .

**Table 2:** Surgical procedure details

	Study group
**FAI etiology**	
**Cam only,** ***n*** **(%)**	30 (86%)
**Pincer only,** ***n*** **(%)**	1 (3%)
**Combined Cam and Pincer,** ***n*** **(%)**	4 (11%)
**Labral treatment**	
**Labral suture (%)**	74 (74%)
**Labrectomy (%)**	26 (26%)

Abbreviation: FAI, femoroacetabular impingement.

Mean age of patients was 38.5 years old (SD: 14.3), with an average time of fluoroscopy of 0.36 minutes (SD: 0.14) (21 seconds). The estimated doses for this population were a DAP of 138.30 cGy/cm ^2^ (SD: 79.39). Therefore, 0.026 mSV of radiation exposure is the equivalent to 1.3 chest X-rays (0.02 mSV).

## Discussion

The present study showed radiation exposure figures of 138.3 cGy cm ^2^ to the operated patient, in line with the previously published. 10 There is a relatively short time of exposure, mean 0.36 minutes in the present study, directly related to the surgical team's experience.

The aim of the team was to perform the minimum radiation exposure to the patients; mean 0.36 minutes, thus mean 21.6 seconds of radiation per surgical procedure. These outcomes agree with previous studies, with 0.32 minutes (19.2 seconds) in average of radiation exposure. 10 In a recent study, Budd et al observed that the mean total fluoroscopy time was 1.10 minutes (66 seconds). 11

Hip arthroscopy complications are mostly regarded to nerve lesions, technical problems during surgery or adhesions, and can reach ranges of 15% complications. The vast majority of complications are solved with no consequences. Most of these complications are related to the placement of portals. 12 A few studies have described the radiation exposure during hip arthroscopy, 10 11 and these focused on a possible relationship between the radiations received in the sex organs and a risk for the future fetus. 9 10 11

Radiation exposure may cause skin lesions and even be incorporated to organs that could suffer changes in cellular deoxyribonucleic acid with a higher risk of dysplasia and carcinogenesis. 11 There is an accepted and published calculation to rule out the skin entrance dose. 13 14 15 Local radiation can cause skin lesions such as erythema, loss of skin or epithelial necrosis with doses from 200 to 1200 cGy cm ^2^ , much higher than the values observed in the present study (138 cGy cm ^2^ ). 8 16

The recently described ultrasound-guided systems are of great interest to reduce these ranges of radiation. 17 18 However, placing the arthroscopic portals with ultrasound is much more challenging than using fluoroscopy in a sterile environment. Given that radiation exposure during hip arthroscopy is 10 to 100 times less than the skin threshold tolerance, fluoroscopy offers a large safety margin. Total radiation during hip arthroscopy procedure is equivalent to 1.3 chest X-rays.

Several limitations have to be considered, although some are clearly inherent to it. The calculated doses are based in theoretical proportions that, very probably, have an interindividual variability based on their adipose tissue thickness, height of the fluoroscopic C-arm, or angulation. However, and besides these limitations, the theoretical calculation gives a very high safety margin.

## Conclusions

Radiation exposure in patients undergoing a hip arthroscopy due to FAI entails figures of 138.3 cGy cm ^2^ on average, corresponding to 1.3 chest X-rays, very low from the safety limits recommended.
